# Beta2-Microglobulin as a Diagnostic Marker in Cerebrospinal Fluid: A Follow-Up Study

**DOI:** 10.1155/2014/495402

**Published:** 2014-05-08

**Authors:** Jana Svatoňová, Klára Bořecká, Pavel Adam, Věra Lánská

**Affiliations:** ^1^Department of Neurology, RHG City Hospital, Mostní 934, 278 01 Kralupy nad Vltavou, Czech Republic; ^2^Department of Clinical Biochemistry, Thomayer Hospital, Vídeňská 800, 140 00 Prague, Czech Republic; ^3^Department of Neurology, Central Military Hospital, U Vojenské nemocnice 1200, 167 00 Prague, Czech Republic; ^4^Department of Neurology of the 3rd Medical Faculty, Charles University, Šrobárova 50, 101 00 Prague, Czech Republic; ^5^Statistical Unit, Institute for Clinical and Experimental Medicine, Vídeňská 1958/9, 140 21 Prague, Czech Republic

## Abstract

Beta2-Microglobulin (**β**2-m) is a low molecular weight protein occurring in all body fluids. Its concentration increases in various pathologies. Increased values in cerebrospinal fluid (CSF) are ascribed to an activation of immune system. Using immunoturbidimetry, we examined concentrations of beta2-microglobulin in cerebrospinal fluid in a large group of 6274 patients with defined neurological diseases. Cell counts, total protein, albumin, glucose, lactic acid, immunoglobulins concentrations, and isofocusing (IEF) were also evaluated. We found substantial changes of CSF **β**2-m concentrations in purulent meningitis, leptomeningeal metastasis, viral meningitis/encephalitis, and neuroborreliosis, while in multiple sclerosis these changes were not significant. Intrathecal synthesis and immune activation were present in these clinical entities. A new normative study enables better understanding of beta2-microglobulin behavior in CSF.

## 1. Introduction

In 1968, Beggard and Bearn first isolated beta2-microglobulin (*β*2-m) from urine of patients with renal proximal tubule disorders. *β*2-m is a small membrane protein (11800 D) that is highly stable during evolution; it is encoded in the sixth chromosome. Beta2-microglobulin is composed of 99 amino acids; due to one peptidic bound it creates a loop. It belongs to the immunoglobulin superfamily, and its primary and secondary structure is strikingly similar to IgG structure; thus, it is suggested to arise from the same ancestral gene [1, 2].

Beta2-microglobulin constitutes the light chain of class I major histocompatibility complex proteins and is, therefore, present on the surface of all nucleated cells. Prevailing expression is on lymphocytes and macrophages, conversely none on erythrocytes [3, 4]. *β*2-m is noncovalently bound to the heavy chain, and during metabolism and degradation it is dissociated and released to all biological fluids. Concentration of beta2-microglobulin reflects a rate of cell membrane renovation and cellular turnover. Production and releasing of *β*2-m are constant and low in healthy people: 0.13 mg/h/kg b.w. *β*2-m is filtered through the glomerulal membrane due to its small size, but then it is nearly completely reabsorbed in the proximal tubules [5].

*Serum and plasma beta2-microglobulin value* increases in many pathologies. *β*2-m is a marker for an activation of the cellular immune system, as well as a tumor marker in certain hematologic malignancies (multiple myeloma, non-Hodgkin lymphoma, Hodgkin's disease, and chronic lymphoblastic leukemia) [6–8]. Serum *β*2-m increases in patients with kidney diseases, because 99% is excreted by the kidneys. *β*2-m can form long protein chains deposited in joints and tissues (dialysis-associated amyloidosis) [9–11].

Increased* urine beta2-microglobulin value* indicates a damage of renal tubules because of the decrease of reabsorption. Measurement of concentrations in both serum and urine enables to differentiate a problem of cellular activation from a renal tubulointerstitial disorder [12–16].

Cerebrospinal fluid (CSF) reflects changes occurring in brain and spinal cord. Unlike blood analysis, examination of cerebrospinal fluid is not disturbed by other organ systems influence, and the CSF compartment is comprehensively autonomous. Assessment of beta2-microglobulin in* cerebrospinal fluid* is recommended within the enhanced level of examination [17]; *β*2-m concentrations in CSF have been proposed as a reliable marker in different inflammatory, autoimmune, or neoplastic central nervous system disorders. Increased CSF values are attributed to an immune activation and lymphoid cell turnover, a production of tumorous cells, and releasing in tissue destruction in CNS.

CSF values of beta2-microglobulin were studied in the context with neurological diseases by many authors. Simultaneous measurement of *β*2-m and ferritin in CSF was found as a useful instrument for differential diagnosis between viral meningitis and bacterial meningitis and for monitoring of ATB therapy effect [18–22]. Many studies confirm significantly elevated CSF *β*2-m values in neonates with cytomegalovirus infection, by reason of an intrathecal synthesis [23–25]. Findings in patients with HIV-1 infection raise a great interest. An elevation of CSF *β*2-m concentration correlates with a disease progression, and a decline of increased value reflects a successful therapy [26–31]. Assessment of specific proteins including beta2-microglobulin is the aim of many studies with dementias like Alzheimer's disease and Parkinson's disease [32–35].

A new study shows an antibacterial role of beta2-microglobulin in further human fluid: the amniotic fluid [36].

A large body of literature on monitoring *β*2-m levels in cerebrospinal fluid is currently available. While most groups of patients described are numerous enough, what is badly needed is a comparison of diagnostical subgroups of defined neurological entities in reasonably large groups. We examined a large CSF sample and follow-up values of beta2-microglobulin in patients with defined CNS diseases to clarify a biological behavior of this marker. We hypothesize that CSF values of *β*2-m would be increased particularly in patients with CNS inflammations and tissue disintegration. Our results support the great importance of *β*2-m examination for differential diagnosis and early therapy of central nervous system diseases, with concordance with the other CSF parameters, incl. CSF cytology, and biochemistry.

## 2. Materials and Methods

In the Laboratory for CSF and Neuroimmunology of the Homolka Hospital (the expert laboratory in CSF analysis in Czech Republic) we evaluated a total of 6274 CSF samples from patients aged from 3 to 86 years. In all the cases, the CSF samples were analysed only for diagnostic purposes, and they were completely investigated. We examinated firstly a CSF cell count (in the Fuchs-Rosenthal chamber) and basic biochemistry including total protein, glucose, lactic acid concentrations, and CEB (coefficient of energy balance) [37]. Secondly, we performed qualitative cytological examination (permanent cytological preparations). Thirdly, albumin CSF/S quotient, IgG, IgA, IgM CSF/S quotients, *β*2-m in CSF and serum, and the isoelectric isofocusing were evaluated within an enhanced CSF protocol [17]. With immunoglobulins, their intrathecal oligoclonal synthesis, if existent, was determined numerically by the formulae according to Reiber [38].

The functional state of blood-CSF barrier could have been expressed by *Q*_alb_ only in the cases when albumin CSF and serum concentrations were measured simultaneously. Evaluation of a blood-CSF barrier permeability is not so important for *β*2-m as for other CSF proteins due to a small molecule of *β*2-m [39]. Nevertheless, physiological CSF concentration of *β*2-m is slightly lower than serum one and *Q*_*β*2-m_ is below 0.8 (see Table 1). Then a blood-CSF barrier impairment may cause an increase in CSF *β*2-m level and an equilibration of CSF and serum concentrations (whether any serum concentration), and consequently *Q*_*β*2-m_ elevated above 0.8. So we can say that only *Q*_*β*2-m_ increased above 1.0 points clearly to the intrathecal synthesis of beta2-microglobulin.

In cytological examination, we used standard and special staining techniques in an effort to detect malignant elements (Toluidine blue stain, Papanicolaou) and the presence of CNS tissue destruction (Oil Red 0) by detecting lipophagic macrophages. Cytological samples were obtained from CSF using a gentle sedimentation technique. Concentrations of *β*2-m were established using immunoturbidimetry in CSF and corresponding sera in Synchron LX-20 analyser. Cytological CSF syndromes with a normal cell count (i.e., up to 10/3 of cells per chamber or up to 4/*μ*L) were classified as normal or pathological oligocytosis (lymphocyte oligocytosis, monocyte oligocytosis, granulocyte oligocytosis, and tumorous oligocytosis) depending on the morphological and functional prevalence of individual cell lines in the cytological picture, whereas, with tumourous oligocytosis, the key factor was the detection of malignant elements in the cytological sample in general. In the case of pleocytosis, an elevated cell count in CSF, cytological syndromes were determined using similar criteria, that is, lymphocyte pleocytosis, monocyte pleocytosis, granulocyte pleocytosis, and tumorous pleocytosis, using classification acc. to Adam et al. [39]. From this point of view, using such a terminology may be often helpful in aetiological identification of the nosological entity involved.

Control group was declared as the group with normal cytological and biochemical findings (including total protein, albumin, immunoglobulins levels, glycorrhachia, and lactic acid concentration, CEB acc. to Bořecká et al. [37]), and after exclusion of the presence of inflammatory or organic neurological impairment. Our study used established laboratory norms—see Table 1. Although these criteria may be considered not to be fully convincing, there is virtually no other way of further differentiation (we do not perform lumbar puncture in healthy people). Samples with parameters not meeting the above criteria were automatically regarded as samples from patients with pathological CSF findings. This large pathological group was divided according to CSF findings into group with multiple sclerosis (*n* = 2674), neuroborreliosis (*n* = 215), brain tumours/malignant infiltrations (*n* = 95), viral meningitis/encephalitis (*n* = 641), bacterial meningitis/encephalitis (*n* = 312), scavenger reaction (*n* = 638), and polyneuritis/polyneuropathies (*n* = 1261). Malignant meningeal infiltration (MMI) or meningeal carcinomatosis is defined as a meningeal metastatic involvement without the presence of lokalized metastases (i.e., meninges are inhabited by malignant elements). This is relatively frequent severe complication of cancer, mostly in secondary, sometimes also in primary tumours of the nervous system. The phagocytosis of a specific substrate, that is, the scavenger reaction or reactions, is characterized by the presence of the phagocytosis of tissue destructive products and cellular detritus. This occurs in the presence of a morphological CNS and PNS impairment, for example, in brain ischemia or in CNS traumas [39].

The values were precisely statistically correlated, leading to substantial concrete findings both in the whole group of patients and after their division according to the presence of normal or pathological biochemical, and even cytological CSF findings. Descriptive methods, Kolmogorov-Smirnov test, box-and-whiskers plots, *t*-test, and one-way ANOVA with pairwise post hoc multiple comparisons were used for statistical analysis. As the *β*2-m distribution is not normal, we performed the logarithmic transformation of *β*2-m for one-way ANOVA testing.

## 3. Results and Discussion

**Figure 1:**
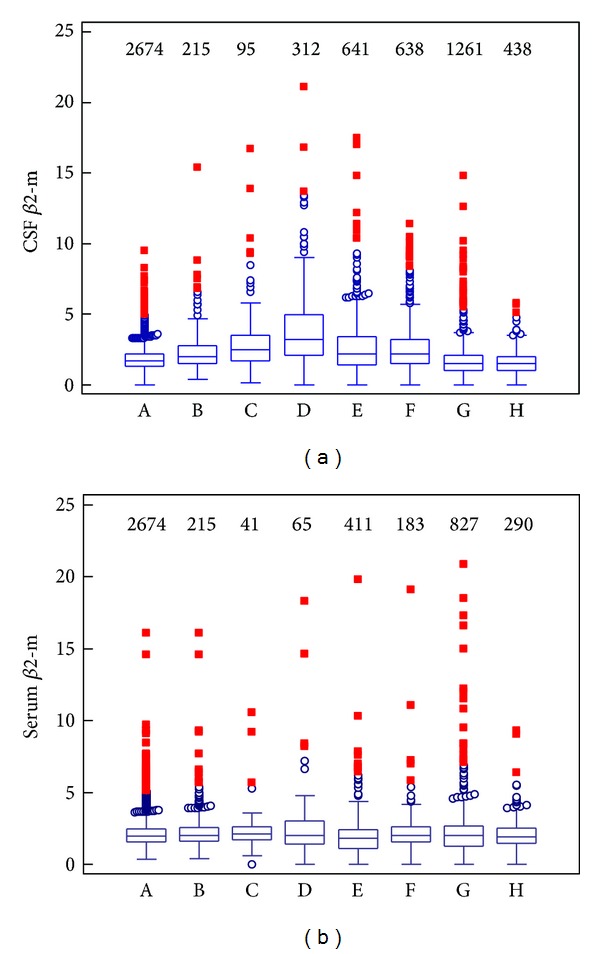
Beta2-microglobulin with diagnostic subgroups: box-and-whiskers graphs. (a) Cerebrospinal fluid beta2-microglobulin. (b) Serum beta2-microglobulin. (A = multiple sclerosis, B = neuroborreliosis, C = brain tumours/malignant infiltrations, D = bacterial meningitis/encephalitis, E = viral meningitis/encephalitis, F = scavenger reaction, G = polyneuritis/polyneuropathies, and H = control group; a number above the box shows sample size).

Results are summarized in Tables 2–4 and Figures 1(a) and 1(b).

As can be seen from Table 2, higher values of *β*2-m in cerebrospinal fluid are present in the pathological group. We found a statistically significant difference between the control group and the pathological group (*P* < 0.001). Conversely, serum *β*2-m values are similar in pathological and control group. Although *β*2-m concentration was not certainly used for determination of the control group, in this group the mean of *β*2-m is in the reference range both in cerebrospinal fluid and in serum. In the pathological group, the mean of *β*2-m is slightly increased and *Q*_*β*2_ is increased only in CSF.

Table 3, Figures 1(a) and 1(b) show values of *β*2-m in CSF and serum with individual diagnostic subgroups. We found the highest median of *β*2-m in cerebrospinal fluid in the group with bacterial meningitis/encephalitis, where increased *β*2-m values are surely caused by inflammatory response and an intrathecal synthesis (*Q*_*β*2_ is 1.39 in this group, when *Q*_*β*2_ > 1.0 means an intrathecal synthesis). The second group is the group with malignant infiltrations or brain tumours; highly increased values of *β*2-m are probably caused by tissue destruction, production of tumorous cells, and inflammatory response as well (*Q*_*β*2_ = 1.32). The third ones are equal groups with viral meningitis/encephalitis and scavenger reaction. We found borderline values of *β*2-m_CSF_ in group with neuroborreliosis. Our results confirm that *β*2-m behaves in cerebrospinal fluid as an inflammatory marker or a tumour marker. Elevated *β*2-m_CSF_ values should lead to a suspicion of CNS inflammation or malignant infiltration, even if no tumorous cells are present in cerebrospinal fluid. On the other hand we demonstrated only slightly or no increased values of CSF *β*2-m and no intrathecal synthesis (evaluated according Q_*β*2_) in groups with multiple sclerosis and polyneuritis/polyneuropathies. Concentrations of CSF *β*2-m probably reflect an activity and a progression of disease in patients with multiple sclerosis. In the control group, we found unique CSF and serum *β*2-m values greater than the upper reference limit (see “maximum” in Table 3); this is because dialysed patients were also included.

In serum, the upper reference limit (3.0 mg/L) was not exceeded by medians in any diagnostic subgroup. Serum *β*2-m values are similar in all groups (see Figure 1(b)).

As can be seen from Table 4, we found a statistically significant difference between the group with bacterial meningitis/encephalitis and the control group (*P* < 0.0001), as well as in comparison with all other groups. In other words, beta2-microglobulin values are the highest in the group with bacterial meningitis/encephalitis (see Table 3 and Figure 1(a)). The difference between groups with bacterial and viral meningitis/encephalitis is very important, because that means the CSF *β*2-m value allows distinguishing between these etiological diagnoses and contributes to early targeted therapy.

Group with the second highest CSF *β*2-m values (see Table 3 and Figure 1(a)), the group of brain tumours and malignant infiltrations, is statistically different from the control group and groups with multiple sclerosis, and polyneuritis/polyneuropathies. But there is no difference among this group and group with viral meningitis/encephalitis, neuroborreliosis, scavenger reaction, that is, values of CSF *β*2-m are similar in these groups.

No difference between the control group and group with polyneuritis/polyneuropathies must be again stressed, highly probably due to the reason, that this diagnostical group is not homogenous neither in clinical findings (concrete neurological peripheral syndromes) nor in laboratory evaluation. The etiology of these units is not usually completely clear, the majority of clinical case is autoimmune or metabolic one. An extensive inflammatory response is unusual in polyneuritis and polyneuropathies.

## 4. Conclusion

Today, laboratories determine, on a routine basis, many protein fractions in CSF and serum (or plasma). Terms like “CSF Proteinic Status” and “CSF proteinogram” appear. We examined a large CSF sample in patients with defined CNS diseases to clarify a biological behavior of beta2-microglobulin. The importance of our study is supported also by the fact that all measurements were performed in one laboratory by the same method of analysis. We found substantial elevation of *β*2-m values in groups with bacterial meningitis/encephalitis, malignant infiltrations or brain tumours, and viral meningitis/encephalitis. In these groups an immune activation or a tissue destruction is present. Our results confirm the role of beta2-microglobulin as an inflammatory or tumorous marker in cerebrospinal fluid. Further studies of beta2-microglobulin in children are needed to clarify a relation with a maturation of the blood-CSF barrier. And it is appropriate to deal with *β*2-m values in further clinical entities (e.g., hereditary degenerative diseases). Measurement of *β*2-m does not need any large costs; a requirement on the CSF amount is minimal. Our results support the great importance of *β*2-m examination for differential diagnosis, early therapy, and monitoring of therapeutic effect of central nervous system diseases.

## Figures and Tables

**Table 1:** Reference ranges of selected parameters of basic CSF examination (determined at our laboratory) [37, 39–41].

Parameter	Range	Unit
Glucose_CSF_	2.20–4.20	mmol/L
Lactate_CSF_	0.7–2.1	mmol/L
CEB (coefficient of energy balance)	28.0–38.0	—
Total protein_CSF_ (TP_CSF_)	0.2–0.4	g/L
Albumin quotient (*Q*_alb_) *Q*_alb_ = [albumin_CSF_]/[albumin_blood_]	to 7.4	×10^*E*^−3^^
Leukocyte count	0–4	/1 µL
*β*2-m_CSF_	0.2–2.0	mg/L
*β*2-m_blood_	1.0–3.0	
*β*2-m quotient (Q_β2_) *Q*_*β*2_ = [*β*2-m_CSF_]/[*β*2-m_blood_]	0.41–0.79	—

**Table 2:** Beta2-microglobulin—comparison between the control group and the pathological group.

Group	N	Beta2-microglobulin (mg/L)		*Q*_β2 _
		Mean CSF	Mean serum	
Control group	438	1.6	2.5	0.64
Pathological group	5836	2.1	2.2	0.95

**Table 3:** Beta2-microglobulin in cerebrospinal fluid with diagnostic subgroups.

Subgroup	N	Beta2-Microglobulin (mg/L)						
		Median	Minimum	Maximum	1st quartile	3rd quartile	2.5th percentile	97.5th percentile
Multiple sclerosis	2674	1.7	0.0	9.5	1.3	2.2	0.8	4.1
Neuroborreliosis	215	2.0	0.4	15.4	1.5	2.8	0.8	6.8
Brain tumours/malignant infiltrations	95	2.5	0.2	16.7	1.7	3.5	0.4	10.1
Bacterial meningitis/encephalitis	312	3.2	0.0	21.1	2.1	4.9	0.6	10.6
Viral meningitis/encephalitits	641	2.2	0.0	17.5	1.4	3.4	0.2	7.4
Scavenger reaction	638	2.2	0.0	24.2	1.5	3.2	0.3	7.6
Polyneuritis/polyneuropathies	1261	1.5	0.0	14.8	1.0	2.1	0.0	4.5
Control group	438	1.5	0.0	5.8	1.0	2.0	0.2	3.4

**Table 4:** CSF Beta2-microglobulin—result of post hoc multiple comparisons of diagnostic subgroups.

Compared groups	Level of Significance
Bacterial meningitis/encephalitis—Control group	*P* < 0.0001
Bacterial meningitis/encephalitis—Viral meningitis/encephalitis	*P* < 0.0001
Bacterial meningitis/encephaliti—Polyneuritis/polyneuropathies	*P* < 0.0001
Bacterial meningitis/encephalitis—Neuroborreliosis	*P* < 0.0001
Bacterial meningitis/encephalitis—Scavenger reaction	*P* < 0.0001
Bacterial meningitis/encephalitis—Multiple sclerosis	*P* < 0.0001
Bacterial meningitis/encephalitis—Brain tumours/Malignant infiltrations	*P* = 0.0066
Brain tumours/Malignant infiltrations—Control group	*P* < 0.0001
Brain tumours/Malignant infiltrations—Multiple sclerosis	*P* < 0.0001
Brain tumours/Malignant infiltrations—Polyneuritis/polyneuropathies	*P* < 0.0001
Viral meningitis/encephalitis—Control group	*P* < 0.0001
Viral meningitis/encephalitis—Multiple sclerosis	*P* < 0.0001
Viral meningitis/encephalitis—Polyneuritis/polyneuropathies	*P* < 0.0001
Neuroborreliosis—Control group	*P* < 0.0001
Neuroborreliosis—Multiple sclerosis	*P* < 0.0001
Neuroborreliosis—Polyneuritis/polyneuropathies	*P* < 0.0001
Scavenger reaction—Control group	*P* < 0.0001
Scavenger reaction—Multiple sclerosis	*P* < 0.0001
Scavenger reaction—Polyneuritis/polyneuropathies	*P* < 0.0001
Multiple sclerosis—Control group	*P* < 0.0001
Multiple sclerosis—Polyneuritis/polyneuropathies	*P* < 0.0001
Other groups	No difference
